# Microbial Properties of Raw Milk throughout the Year and Their Relationships to Quality Parameters

**DOI:** 10.3390/foods11193077

**Published:** 2022-10-04

**Authors:** Huizhi Yuan, Sufang Han, Shufei Zhang, Yuling Xue, Yaoguang Zhang, Han Lu, Shijie Wang

**Affiliations:** 1 College of Food Science and Biology, Hebei University of Science and Technology, Shijiazhuang 050018, China; 2 Junlebao Dairy Group Co., Ltd., Shijiazhuang 050221, China

**Keywords:** raw milk, microbiota, quality parameters, titratable acidity, Chinese region, diversity index

## Abstract

Raw milk microbiota is complex and influenced by many factors that facilitate the introduction of undesirable microorganisms. Milk microbiota is closely related to the safety and quality of dairy products, and it is therefore critical to characterize the variation in the microbial composition of raw milk. In this cross-sectional study, the variation in raw milk microbiota throughout the year (*n* = 142) from three farms in China was analyzed using 16S rRNA amplicon sequencing, including α and β diversity, microbial composition, and the relationship between microbiota and milk quality parameters. This aimed to characterize the contamination risk of raw milk throughout the year and the changes in quality parameters caused by contamination. Collection month had a significant effect on microbial composition; microbial diversity was higher in raw milk collected in May and June, while milk collected in October and December had the lowest microbial diversity. Microbiota composition differed significantly between milk collected in January–June, July–August, and September–December (*p* < 0.05). Bacterial communities represented in raw milk at the phylum level mainly included Proteobacteria, Firmicutes and Bacteroidota; *Pseudomonas*, *Acinetobacter*, *Streptococcus* and *Lactobacillus* were the most common genera. Redundancy analysis (RDA) found strong correlations between microbial distribution and titratable acidity (TA), fat, and protein. Many genera were significantly correlated with TA, for example *Acinetobacter* (R = 0.426), *Enhydrobacter* (R = 0.309), *Chryseobacterium* (R = 0.352), *Lactobacillus* (R = −0.326), norank_o__DTU014 (R = −0.697), *norank_f__SC-I-84* (R = −0.678), and *Subgroup_10* (R = −0.721). Additionally, *norank_f__ Muribaculaceae* was moderately negatively correlated with fat (R = −0.476) and protein (R = −0.513). These findings provide new information on the ecology of raw milk microbiota at the farm level and contribute to the understanding of the variation in raw milk microbiota in China.

## 1. Introduction

Raw milk is rich in nutrients and provides ideal nutritional conditions for many microorganisms [1,2]. Milk should be sterile within the healthy udder cells, and when it leaves the udder, it usually collects small amounts of microorganisms (mainly lactic acid bacteria), but possible exposure to exogenous contaminants furthers a complex microbiota originating from different sources [3,4], the most common being the udder and teat surface [5]. In addition, the number and type of microorganisms in raw milk are influenced by many factors, such as milking equipment cleanliness, season, water, feed, and animal health [4]. It is important to understand the factors that positively or negatively affect the microbiota of raw milk because this affects the safety and quality of the food produced.

The hygienic production of milk is a major challenge for the global dairy industry [6]. With the increasing demand for dairy products, bacterial contamination has become a worldwide concern. The microbial content of milk is the main characteristic that determines its quality [7], as well as the sensory and quality characteristics of dairy products [8,9]. The common genera found in milk include *Lactobacillus*, *Streptococcus*, *Enterococcus*, and a subset of important psychrophilic bacteria, usually including *Pseudomonas* and *Acinetobacter* [10,11]. Psychrophilic bacteria can grow and proliferate at low temperatures and spoil milk by producing extracellular lipases and proteases [12]. In addition, common foodborne pathogens (e.g., *Staphylococcus aureus*, *Listeria monocytogenes*, and *Salmonella* spp.) may be present in milk, and the use of raw milk contaminated with these bacteria can lead to the development of various foodborne diseases, posing a potential risk to human health [3,13].

In addition, milk quality depends on chemical parameters (fat and protein content), total bacterial count (TBC), and somatic cell count (SCC) [14]. Fat and protein concentration determines the price of the product and health of the herd [15]. SCC is an important parameter for udder health and has become the gold standard for milk quality [16], while TBC is used to assess the quality and safety of raw milk [17]. High SCC and TBC in milk may lead to the production of enzymes that degrade fat and protein, reducing the quality of the milk and its products [18]. These parameters may be influenced by the environment, milking practices, and udder hygiene, determining the safety and hygiene of the final product [19].

Many studies on milk microbiota have focused on aspects related to udder and animal health rather than milk safety and quality [5]. Although the high diversity of milk microbiota has been described in several studies, most of the studies on longitudinal variation in microbiota have focused on seasonal divisions [6,20], while few studies focused on a more precise monthly timescale. In this study, the microbiota of raw milk from three dairy farms in Shijiazhuang, China, were examined using 16s rRNA technology to characterize the major microorganisms present in raw milk and to analyze the patterns of variation throughout the year and correlations with milk quality parameters (TA, TBC, SCC, milk fat and protein). This provides the basis for good hygiene practices and standardized operational procedures in milk production to provide high quality dairy products.

## 2. Materials and Methods

### 2.1. Sample Collection

Between September 2020 and August 2021, 142 raw milk samples were collected from three farms in Shijiazhuang, China. Each week, 15 mL of milk sample was collected from milk storage tanks at each farm and stored in 50 mL sterile centrifuge tubes. Samples were transported to the laboratory on ice and stored at −80 °C for approximately 4 months prior to analysis. Junlebao Dairy Group Co., Ltd. (Shijiazhuang, China) provided milk samples and experimental equipment in this study.

### 2.2. DNA Extraction

First, 200 mg of each sample was weighed into a 2 mL centrifuge tube and centrifuged at 12,000 rpm for 5 min; the supernatant was retained, and the fat removed. Bacterial genomic DNA was extracted using the Milk Bacteria DNA Extraction Kit and stored at −20 °C until analysis [21]. The concentration of extracted DNA was determined using the NanoDrop ND-1000 (Thermo Fisher Scientific, Cleveland, OH, USA) for amplification.

### 2.3. PCR and 16S rRNA Gene Sequencing

The V3–V4 region of the bacterial 16S rRNA gene was amplified using primers 341F and 805R. The PCR cycle parameters were as follows: 1 min at 98 °C; 10 cycles of 15 s at 98 °C, 15 s at 58 °C, and 15 s at 72 °C; followed by a final extension step for 5 min at 72 °C.

The 16S rRNA gene amplicons were sequenced using the Illumina NovaSeq instrument by Shanghai Weihuan Biotechnology Co., Ltd. (Shanghai, China). Overlapping paired-end 16S rRNA sequence reads were processed with DADA2 using QIIME2 (release 2020.2) [22]. Unique amplicon sequence variants (ASVs) were assigned a taxonomy and aligned to the SILVA reference database at 99% sequence similarity [23,24].

### 2.4. Microbial Data Pre-Processing

Data analysis was performed in R [25]. Preliminary preprocessing of ASV tables was performed using the Phyloseq package [26]. The VennDiagram package was used to obtain information on species richness and evenness within samples, as well as information on common and unique ASVs across samples [27]. The vegan package was then used to analyze the community structure differences among samples and subgroups using Principal coordinate analysis (PCoA) [28]. Next, histograms of microbial composition were plotted using the ggplot2 package to characterize the relative abundance of major microorganisms in raw milk. In addition, the ggridges package was used to demonstrate the possible presence of different microbial contaminants in raw milk from different sampling periods, and finally RDA was performed to explore the correlation between microbial composition of raw milk and quality parameters.

### 2.5. Statistical Analysis

The Chao1, ACE, Shannon, and Simpson index were used to assess the alpha diversity of the bovine milk microbiota in different months. Statistical differences were identified using *t*-tests or Wilcoxon rank sum tests. The Adonis statistical analysis method was chosen to test the significance of differences in community structure of grouped samples. The Kruskal–Wallis test was used to analyze differences in the relative abundance of major phyla and genera (relative abundance > 1%) by month. Correlation coefficients between the major genera and milk quality parameters were analyzed using the Spearman’s rank correlation method in the R. *p* < 0.05 was considered statistically significant.

## 3. Results

### 3.1. Sequence Quality Control

A total of 18240516 (461-3511279) high quality 16s rRNA gene sequences were obtained using 16S rRNA sequencing. A total of 84834 ASVs (2217-11859) were finally obtained using a 99% sequence similarity threshold. The Good’s coverage was 99.94% on average (99.73–99.99%), further indicating that it was sufficient for bovine milk microbiota analysis at the current sequencing depth.

### 3.2. Alpha and Beta Diversities of Milk Microbiota Based on Months

**Figure 1:**
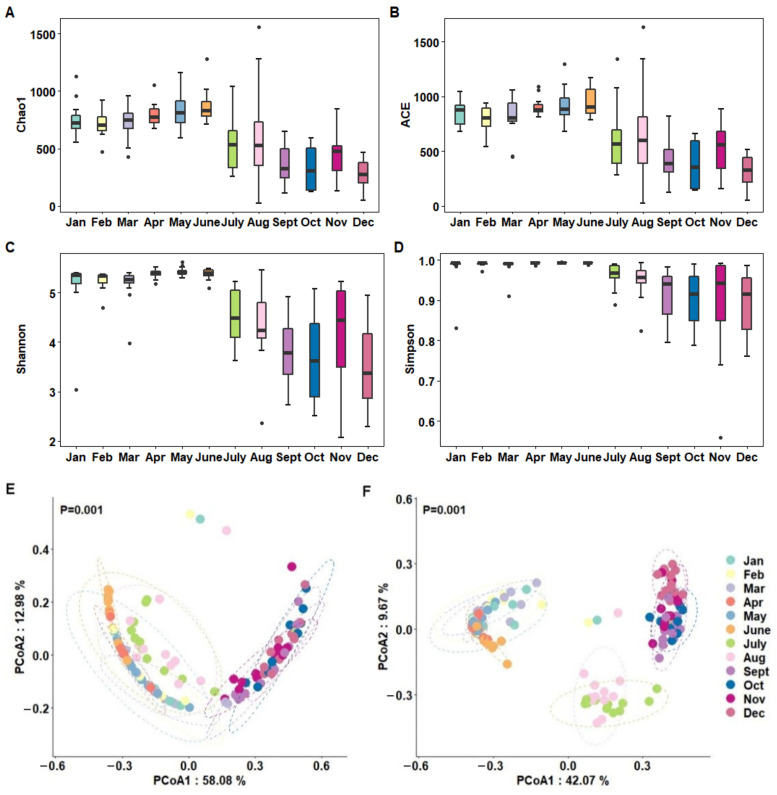
Microbial diversity analysis of raw milk throughout the year. (**A**–**D**) α-diversity of milk microbiota (Chao1, ACE, Shannon, and Simpson indexes) throughout the year. (**E**,**F**) PCoA of the bacterial structure of milk microbial communities at the phylum (**E**)/genus (**F**) level throughout the year. In E and F, dots represent the samples with different colors indicating the month of collection, while the dotted lines represent 95% confidence ellipses. The horizontal and vertical axes represent the first and second principal coordinates, respectively, and the percentages on the horizontal and vertical axes are the contribution of that principal coordinate to the difference of the sample matrix data. The closer the projection distance between two points on the coordinate axis, the more similar the community composition.

Box plots of the α-diversity analysis showed the distribution of diversity indices among samples collected throughout the year (Figure 1A–D). Overall, bacterial community richness and diversity were generally higher in spring samples and lower in autumn samples, whereas they were more variable in summer and winter samples, with fluctuation points in June and December, respectively. When grouped by month, raw milk species richness and diversity were higher from May to June and lower in October and December (Table 1).

Changes in the microbial composition of raw milk in different months were compared by β-diversity analysis (Figure 1E,F). The results showed that the microbial composition of raw milk from January to August differed significantly at the phylum level (*p* = 0.001) from September to December, with a more dispersed distribution of samples in July and August. PCoA at the genus level showed significant differences in microbial community composition between the three groups from January to June, July to August and September to December (*p* = 0.001). When raw milk samples were grouped according to sampling season, the clustering results were more dispersed for both summer and winter samples, while the clustering effect was more pronounced for both spring and autumn samples, implying that the bacterial communities differed more between summer and winter samples and less between spring and autumn samples.

### 3.3. Differences in Microbial Composition of Raw Milk between Samples Collected in Different Months

**Figure 2:**
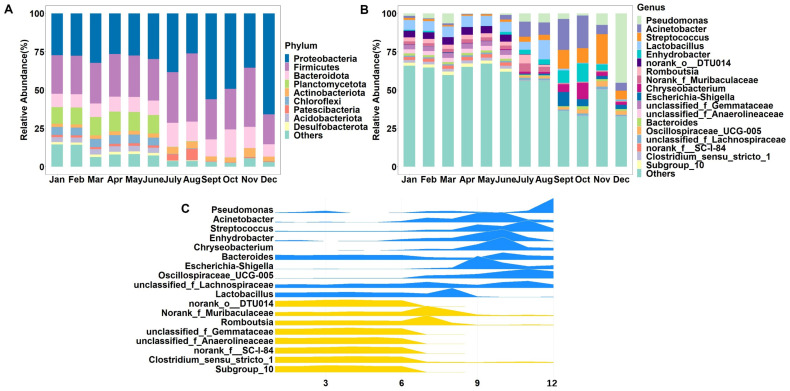
Relative abundance of bacterial phyla (**A**)/genera (**B**) in raw milk according to month. Bacterial genera with a relative abundance of <1% were classified as “others”. (**C**) Abundance distribution of ASV in raw milk throughout the year.

At the phylum level (Figure 2A), Proteobacteria (36.84% ± 13.20%), Firmicutes (28.91% ± 6.72%), and Bacteroidota (11.24% ± 3.20%) were the dominant phyla, with relative abundance accounting for more than 70% of the total bacteria for the year. There were significant differences in the microbial composition of milk in different months, with Planctomycetota and Chloriflexi being more abundant from January to June. Bacteroidota was more abundant in July and October, *Actinobacteria* and Firmicutes in July, August and November, and Proteobacteria in September and December.

The most common genera (Figure 2B) in raw milk were *Pseudomonas* (6.90% ± 12.34%), *Acinetobacter* (6.42% ± 7.41%), *Streptococcus* (5.05% ± 5.83%) and *Lactobacillus* (5.02% ± 3.74%). The relative abundance of these four genera varied significantly between seasons, with the highest relative abundance of bacteria in spring and summer samples being *Lactobacillus*, in autumn samples being *Acinetobacter* and *Streptococcus*, and in winter samples being *Pseudomonas*. In terms of months, the relative abundance of *Lactobacillus* was higher from January to August, that of *Acinetobacter* was higher in July, September and October, and that of *Streptococcus* was higher from September to December. *Chryseobacterium* had the highest abundance from September to October, and *Pseudomonas* showed the highest abundance from November to December.

Next, the temporal dynamics of the raw milk microbiota were investigated in order to characterize the potential for contamination by different microorganisms throughout the year. The abundance of important bacterial ASVs in raw milk was expressed in relation to the sampling period using ridgeline plots (Figure 2C), focusing on highlighting the dynamics of microbial enrichment at specific times. The results show that *Pseudomonas* abundance increased sharply in November, *Acinetobacter* in June and August, *Streptococcus* in August and October, and *Enhydrobacter*, *Chryseobacterium*, and *Escherichia-Shigella* all increased in August, while *Bacteroides* and *unclassified_f _Lachnospiraceae* varied more moderately throughout the year. In addition, *Lactobacillus* abundance decreased in August. Norank_o__DTU014 and other genera shown in yellow have a higher probability of contaminating raw milk in December (Figure 2C).

### 3.4. Correlation Analysis between Dominant Bacterial Genera and Raw Milk Quality Parameters

**Figure 3:**
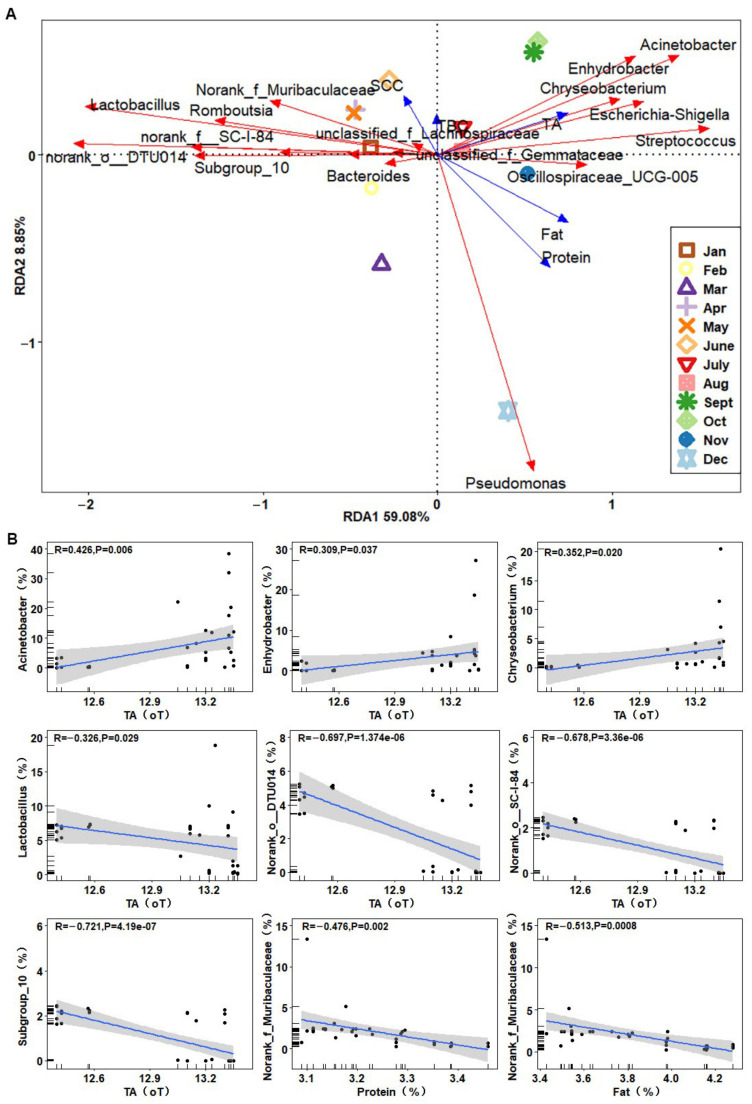
Correlation analysis between the top 20 bacterial genera and quality parameters. (**A**) RDA between the relative abundances of bacteria genera and quality parameters. Quality parameters (environmental factors) are represented by blue arrows, with samples represented by points of different shapes, while the genera are represented by vectors (red arrows). The length of the line between the arrow and the origin represents the magnitude of the correlation between a given environmental factor and the distribution of communities and species. The angle between the arrows indicates the correlation, with acute angles indicating positive correlation and obtuse angles indicating negative correlation. (**B**) Scatter plots showing significant correlations. The shaded area represents the 95% confidence interval.

The overall RDA model with protein, fat, TBC, SCC, and TA as explanatory variables was significant (*p* = 0.018); of the overall variation in taxon composition, RDA1 and RDA2 explained 59.08% and 8.85% of the total variation, respectively. Figure 3 highlighted the top 20 genera significantly associated with one or both of the first two RDA axes, of which *Acinetobacter*, *Enhydrobacter*, *Chryseobacterium*, *Lactobacillus*, and norank_o__DTU014 contributed significantly to the differences in the bacterial fractions of raw milk.

TA, fat, and protein showed strong correlations with microbial distribution in the samples. Raw milk from September to November had higher acidity, and milk in December was higher in protein and fat. Significant correlations were found between many genera and TA; *Acinetobacter* was moderately positively correlated with TA (R = 0.426), *Enhydrobacter* (R = 0.309) and *Chryseobacterium* (R = 0.352) were weakly positively correlated with TA, and *Lactobacillus* was weakly negatively correlated with TA (R = −0.326), norank_o__DTU014 (R = −0.697), *norank_f__SC-I-84* (R = −0.678) and *Subgroup_10* (R = −0.721) were strongly negatively correlated with TA. In addition, *norank_ f_ Muribaculaceae* was moderately negatively correlated with fat (R = −0.476) and protein (R = −0.513).

## 4. Discussion

The microbiota composition of raw milk varies considerably at different times of the year. This study shows that seasonality contributes up to 10% of the variation in the microbiota composition of raw milk, with higher α-diversity in samples collected in the spring/summer [29]. In this paper, samples collected in May and June had higher species richness and diversity, which is consistent with previous studies. Although there are multiple possible explanations for the increase in species richness, this finding is consistent with a significant increase in the relative abundance of Planctomycetota. The increase in species richness observed in May and June may be due to seasonal changes, such as increased precipitation, that promote the growth and transfer of Planctomycetota to the udder surface [30]. Cow feeding practices may also had an effect [31]; Cows that graze outdoors in the summer have significantly higher total bacterial counts on the teat surface, so special attention needs to be paid to the hygiene of the teat surface during milking.

Furthermore, the bacterial community of raw milk samples obtained during different sampling seasons could be divided into two or three distinct communities. Notably, the bacterial community structure in July and August was similar to that of January to June at the phylum level, whereas it was highly specific at the genus level. Raw milk collected in July and August was exposed to greater bacterial variation. Similarly, Liang et al. [32] found greater variation in bacterial communities in summer samples. However, a study conducted in Ireland showed that samples collected in November had the greatest diversity and unique microbiota composition compared to samples collected in April and August [29]. Ireland has a temperate maritime climate with high levels of annual precipitation, while Hebei has a temperate continental monsoon climate [32]. Thus, differences in climatic conditions and geographical factors may explain the differences in the bacterial composition of raw milk.

The microbial composition at the phylum level in this study was similar to that in the previous studies [6,33], with Proteobacteria, Firmicutes, and Bacteroidota being the main phyla in raw milk. Compared to other studies, Planctomycetota were more abundant in this study, especially from January to June. They are commonly attached to surfaces in various environments, but their exact role remains uncertain [34]. Genera commonly found in raw milk, such as *Pseudomonas*, *Acinetobacter*, *Lactobacillus* and *Streptococcus*, were also detected in our study. *Pseudomonas* was more prevalent in autumn and winter, *Acinetobacter* was more abundant in summer and autumn, *Streptococcus* had the highest relative abundance in autumn, and *Lactobacillus* had the lowest relative abundance in autumn. These results suggest that there is seasonal variation in all major bacteria in milk. To better understand the relevance of microorganisms to different sampling periods, we tracked the trends in relative abundance of different microorganisms with sampling period, which was used to predict the possibility of microorganisms contaminating raw milk during different sampling periods. The ridgeline plot is a localized over-lapping density plot that is useful for visualizing changes in distribution over time. Raw milk collected in August and December was more susceptible to microbial contamination compared to other times of the year, and the types of microorganisms contaminating raw milk differed between the 2 months. In contrast, common bacteria that are considered universal hygiene indicators [35], including *Lactococcus* and *Bacillus* had relative abundances of <1% in this study. This is supported by studies showing that the relative abundance of *Lactococcus* was lower in healthy milk and bulk canned milk but higher in mastitis-affected milk [36]. Higher levels of *Bacillus* in raw milk are usually associated with temperature abuse in bulk tanks, where *Bacillus* endospores can survive autoclaving, allowing them to persist and potentially cause milk spoilage [37,38].

Milk is maintained at refrigerated temperatures until collection and processing, and both lower storage temperatures and large temperature fluctuations promote the growth of Psychrophilic bacteria, such as *Pseudomonas* and *Acinetobacter* [39]. *Pseudomonas* is closely associated with food spoilage and is currently considered one of the main causes of microbial contamination of raw milk [40]. Studies have observed the presence of *Acinetobacter* and *Chryseobacterium* in milk, which may be the result of environmental or teat skin contamination [41]. Udder health can influence the abundance of *Streptococcus* in milk [42], but the relative abundance of *Streptococcus* was not associated with increased SCC or TBC in milk in this study, suggesting that these *streptococci* may not be from infected udders. Studies have shown that milking equipment is an important source of psychrophilic bacteria in raw milk. Therefore, maintaining the hygiene of milking equipment can reduce the impact of Psychrophilic bacteria on raw milk i.e., increased acidity. In addition to this, raw milk microbiota contains high levels of *Lactobacillus* that reduce the growth of harmful microorganisms in milk, which can lead to milk spoilage and disease [43].

Milk quality is an important issue for the dairy industry and monitoring bacteria associated with raw milk quality is imperative [44]. Increased acidity can be caused by bacterial formation in raw milk, including *Chryseobacterium*, *Enhydrobacter*, and *Acinetobacter* associated with milk storage temperature and environment. RDA didn’t identify genera associated with TBC; furthermore, bacterial colonization of the mammary ducts or teat skin of healthy cows neither significantly affected the total number of bacteria in the milk nor the bacterial counts during refrigerated storage. Celano et al. [45] found that Xanthomonadaceae, Enterobacteriaceae, and Pseudomonadaceae were positively correlated with SCC and hypothesized that higher abundances of these pathogenic organisms in winter were associated with the possible onset of mastitis. In this study, *Candidatus_Saccharimonas* was positively correlated with SCC (data not shown); however, as far as a search of the literature shows, there are no studies on *Candidatus_Saccharimonas* in raw milk or its possible association with mastitis. Studies show that cattle manure is rich in *Candidatus_Saccharimonas*, which produces acid-promoting metabolites that lower pH as its relative abundance increases [46]. *Candidatus_Saccharimonas* is a conditionally pathogenic bacterium that is significantly elevated in the gut of patients with gout [47]. In addition, Guo et al. [46] detected higher abundance of *Candidatus_Saccharimonas* in the rumen microbiota of cows with vertebral plate infection. This suggests that it might be possible for *Candidatus_Saccharimonas* to cause mastitis, a hypothesis that is worthy of future study

Milk provides various nutrients that help to maintain health and normal growth. In this study, milk collected in December contained higher levels of milk fat and protein, while these were lower in milk collected in June. Similarly, Celano et al. [45] found that milk lipid and protein levels were lower in summer samples and higher in winter samples. These differences between collection months may be related to diet, intestinal absorption and environmental factors. *Norank_f_Muribaculaceae* may be present in healthy intestine [48], and it was associated with the distribution of nutrients in milk in this study. In a similar vein, Hou et al. found that *norank_f_Muribaculaceae* was negatively correlated with all indicators related to obesity [49]. Furthermore, this study found that *norank_f_Muribaculaceae* was positively correlated with milk yield, suggesting that it could be used as a bacterial biomarker for milk synthesis [50].

## 5. Conclusions

In summary, this study compared the bacterial diversity in raw milk collected throughout the year and explored the bacteria that may contaminate raw milk at different periods, the possible sources of these bacteria, and the correlation with raw milk quality parameters. Our results clearly show that raw milk collected during different months has highly variable microbiota, especially in July and August. Raw milk collected in August and December had a higher probability of microbial contamination. The microbial compo-sition of raw milk is closely related to TA, fat, and protein content, so changes in milk quality parameters can also change microbiota composition. This study may help to control the risk of microbial contamination at different times of year.

## Figures and Tables

**Table 1:** Bacterial richness and diversity indexes.

Month	Chao ^1^	ACE	Shannon	Simpson
Jan	755.69 ± 45.64 ^ab^	846.90 ± 34.25 ^ab^	5.12 ± 0.19 ^b^	0.98 ± 0.01 ^b^
Feb	708.80 ± 32.51 ^ab^	797.71 ± 33.77 ^ab^	5.24 ± 0.06 ^ab^	0.99 ± 0.002 ^ab^
Mar	728.85 ± 44.85 ^ab^	809.75 ± 56.20 ^ab^	5.16 ± 0.11 ^b^	0.98 ± 0.01 ^b^
Apr	794.71 ± 30.32 ^a^	906.28 ± 25.24 ^a^	5.39 ± 0.03 ^ab^	0.99 ± 0.00 ^ab^
May	831.98 ± 46.90 ^a^	916.97 ± 50.83 ^a^	5.43 ± 0.03 ^a^	0.99 ± 0.00 ^a^
June	870.34 ± 44.58 ^a^	942.46 ± 37.77 ^a^	5.38 ± 0.03 ^ab^	0.99 ± 0.00 ^ab^
July	525.98 ± 67.74 ^bc^	617.68 ± 92.42 ^bc^	4.49 ± 0.16 ^c^	0.96 ± 0.01 ^c^
Aug	631.03 ± 129.00 ^b^	692.62 ± 134.51 ^b^	4.29 ± 0.22 ^c^	0.95 ± 0.01 ^c^
Sept	354.40 ± 52.52 ^bc^	424.13 ± 66.44 ^bc^	3.82 ± 0.22 ^c^	0.91 ± 0.19 ^c^
Oct	323.12 ± 58.78 ^c^	375.38 ± 68.90 ^c^	3.67 ± 0.28 ^c^	0.90 ± 0.02 ^c^
Nov	444.40 ± 53.90 ^bc^	523.92 ± 65.27 ^bc^	4.18 ± 0.30 ^c^	0.90 ± 0.04 ^c^
Dec	268.13 ± 37.29 ^c^	313.85 ± 43.82 ^c^	3.54 ± 0.26 ^c^	0.90 ± 0.22 ^c^

^1^ Results are expressed as mean ± SD. Data with different letters (^a^, ^b^, ^c^) in one column are significantly different (*p* < 0.05).

## Data Availability

The data presented in this study are available on request from the corresponding author.

## Abbreviations

RDA	redundancy analysis
PCoA	Principal coordinate analysis
ASVs	amplicon sequence variants
TA	titratable acidity
TBC	total bacteria count
SCC	somatic cell count
